# Effects of fish stocking and removal on plankton communities and trophic status in deep alpine-type lakes of the Five Lakes Valley (Tatra Mountains): Bottom-up and top-down regulation in the ecology of offshore animals. The lifetime passion of Z.Maciej Gliwicz

**DOI:** 10.1093/plankt/fbag016

**Published:** 2026-03-26

**Authors:** Mirosław Ślusarczyk, Tomasz Brzeziński, Piotr Dawidowicz, Andrzej Kapusta, Tomasz Zwijacz-Kozica, Tomasz Kuczyński, Agnieszka Ochocka, Krzysztof Kozłowski

**Affiliations:** Department of Hydrobiology, Institute of Ecology, Faculty of Biology in Biological and Chemical Research Centre of University of Warsaw, Żwirki i Wigury 101, 02-089 Warszawa, Poland; Department of Hydrobiology, Institute of Ecology, Faculty of Biology in Biological and Chemical Research Centre of University of Warsaw, Żwirki i Wigury 101, 02-089 Warszawa, Poland; Department of Hydrobiology, Institute of Ecology, Faculty of Biology in Biological and Chemical Research Centre of University of Warsaw, Żwirki i Wigury 101, 02-089 Warszawa, Poland; National Inland Fisheries Research Institute, Oczapowskiego 10, 10-719 Olsztyn, Poland; Tatra National Park, Kuźnice 1, 34-500 Zakopane, Poland; Maritime Institute, Gdynia Maritime University, Roberta de Plelo 20, 80-548 Gdańsk, Poland; Department of Aquatic Ecosystems Research, Institute of Environmental Protection—National Research Institute, Słowicza 32, 02-170 Warszawa, Poland; Department of Ichthyology and Aquaculture, University of Warmia and Mazury in Olsztyn, Oczapowskiego 5, 10-719 Olsztyn, Poland

**Keywords:** alpine lakes, fish stocking and eradication, zooplankton community shift, trophic cascade, ecosystem restoration, water transparency

## Abstract

Most alpine-type lakes in the Tatra Mountains were stocked with salmonid fish during the 19th and 20th centuries, triggering profound ecological shifts. In the Five Lakes Valley, trout introductions into Przedni Staw Polski, Czarny Staw Polski and Wielki Staw Polski had contrasting effects on pelagic ecosystems. In the smaller and shallower Przedni Staw Polski and Czarny Staw Polski, fish introduction was associated with the loss of large-bodied zooplankton, increased trophic status and a rapid decline in water transparency—from 13–18 m to 5–7 m. Between 2021 and 2023, partial fish removal in Przedni Staw Polski resulted in a marked increase in water transparency (from 7 to 17 m), coinciding with reduced surface phytoplankton biomass. This improvement likely reflected decreased phosphorus input from fish excretion rather than enhanced zooplankton grazing. Large-bodied cladocerans did not recolonize the lake, although small-bodied taxa increased in abundance. In contrast, Czarny Staw Polski—where fish remain abundant—showed no signs of recovery: water transparency declined further and zooplankton community structure remained unchanged. Unlike the other stocked lakes, Wielki Staw Polski—the largest and deepest—retains high transparency and large-bodied zooplankton, likely due to low fish density or availability of predator-free refugia. While partial fish control in Przedni Staw Polski yielded rapid benefits, restoring natural ecosystem functioning in these fragile alpine ecosystems will require complete fish eradication.

## INTRODUCTION

High-mountain lakes exhibit a unique set of physical and ecological features that distinguish them from lowland lakes. Their relatively small catchment areas, combined with steep, rocky and soil-free slopes, strongly limit the influx of nutrients from the surrounding landscape. These constraints are further compounded by high precipitation and rapid water turnover, which promote continuous nutrient flushing from the system. As a result, such lakes typically exhibit extremely low nutrient concentrations, low biological productivity and simplified food webs. Their biocenoses are further shaped by harsh climatic conditions (short growing seasons and low water temperatures), which slow the pace of natural ecological succession and limit species richness (Karlsson *et al.,* 2005; Catalan *et al.*, 2006; Moser *et al.*, 2019).

Importantly, high-altitude lakes are typically naturally fishless due to physical barriers that prevent fish colonization. In these systems, the absence of visually hunting apex predators such as fish allows zooplankton communities to be dominated by large-bodied species, which are more effective at grazing phytoplankton than smaller-bodied forms (Gliwicz, 1990, 2003). As a consequence of widespread fish introductions, such pristine food webs are rare in human-impacted landscapes.

Over the past centuries, anthropogenic pressures have gradually expanded from lowland regions into high-mountain environments. In earlier periods, deforestation and sheep grazing played a major role in intensifying nutrient inputs from watersheds into previously oligotrophic lakes (Likens and Bormann, 1974; Bajard *et al.*, 2018; Haliuc *et al.*, 2020). In more recent decades, increasing recreational use of mountain areas and, in particular, the artificial stocking of fish into naturally fishless alpine lakes have become the dominant pressures (Ventura *et al.*, 2017). This latter practice, largely motivated by recreational fishing interests, has profoundly disrupted native food webs, sometimes leading to prominent alterations of the biogeochemical cycling of nutrients (Dawidowicz, 1985; Schindler *et al.*, 2001).

Fish exert both direct and indirect effects on nutrient dynamics and, consequently, on primary productivity in these systems. On the one hand, their predation on large-bodied zooplankton reduces grazing pressure on phytoplankton, often leading to increased proliferation of algal biomass and reduced water transparency (Carpenter *et al.*, 1985; Schindler *et al.*, 2001). On the other hand, fish can stimulate primary productivity by facilitating internal nutrient loading through sediment resuspension (Vanni, 2002), mineralization of allochthonous inputs such as terrestrial insects (Schindler *et al.*, 2001), and by reducing nutrient export via suppression of aquatic insect emergence, which would otherwise transfer nutrients out of the system (Hurlbert *et al.*, 1972; Pope *et al.*, 2009; Wesner, 2016). As a result, fish introduction may increase primary production, often accompanied by increased algal biomass, changes in community composition and the loss of large-bodied zooplankton (Brooks and Dodson, 1965; Carpenter *et al.*, 1985; Ventura *et al.*, 2017).

The first known attempts to stock Tatra lakes date back to the 19th century and involved several lakes in the Gąsienicowa Valley (Gliwicz, 1985). In one of the earliest documented cases of fish-induced ecological restructuring, a dramatic shift in the plankton community of Zielony Staw Gąsienicowy (Green Lake) was observed following the introduction of trout. Large-bodied zooplankton species were rapidly replaced by smaller taxa. These observations, made by a young researcher, Gliwicz (Gliwicz, 1963), anticipated the now classic demonstrations by Hrbácek *et al.* (*Hrbácek et al.*, 1961) and Brooks and Dodson (Brooks and Dodson, 1965) of the pivotal role of fish in shaping lake food webs. Gliwicz’s early work not only captured the essence of trophic cascades before the term was coined but also highlighted the vulnerability of fishless alpine lakes to anthropogenic disturbance—a contribution that remains foundational in modern limnology.

**Fig. 1:**
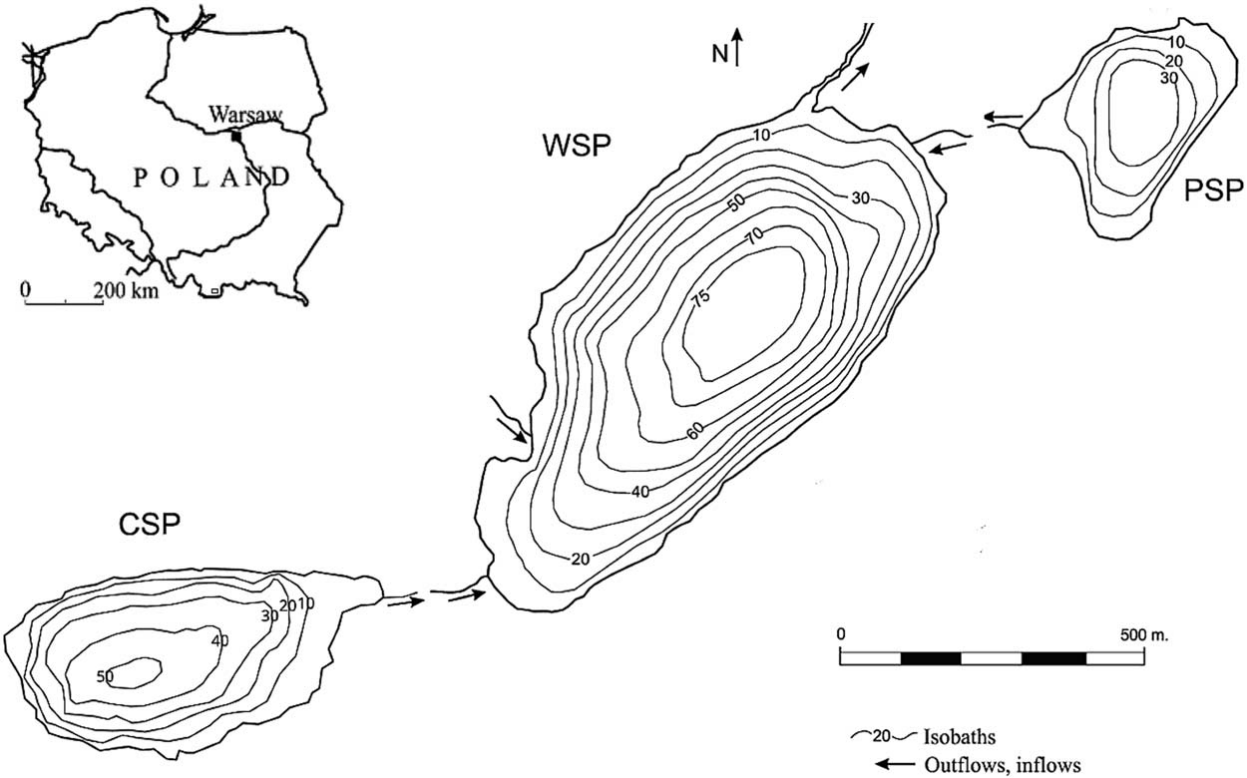
Bathymetric maps of the studied lakes in Dolina Pięciu Stawów (Five Lakes Valley) in the Tatra Mountains, south of Poland: Przedni Staw Polski (PSP), Czarny Staw Polski (CSP) and Wielki Staw Polski (WSP).

Gliwicz’s warnings soon proved prophetic when, a few years later, fish were introduced into nearly all major lakes of the neighboring Five Lakes Valley (Dolina Pięciu Stawów, Fig. 1), leading to profound and lasting ecological consequences. Prior to the establishment of Tatra National Park in 1955 and subsequent fish introductions in the 1960s, these lakes already exhibited an altered trophic state associated with nutrient inputs from grazing livestock (Fig. 1).

Following the Park’s creation and the prohibition of sheep grazing, water quality temporarily improved and water transparency increased, likely reflecting a decline in external nutrient inputs. This favorable trend, however, was not observed in Przedni Staw Polski (PSP), where the construction of a large mountain shelter on its shore in the mid-1950s markedly increased tourism pressure within the catchment, with clear implications for the lake’s PSP trophic state. Subsequent unauthorized fish introductions, initiated under unclear circumstances following the shelter opening, further altered the trophic status of these formerly fishless lakes of the valley (Gliwicz, 1985; Sienkiewicz and Gąsiorowski, 2016).

The ecological impacts of fish introductions varied among lakes, depending on lake morphology and exposure to tourism. The most rapid and pronounced decline in water transparency occurred in PSP—the smallest and the most accessible of the three lakes in the valley. Czarny Staw Polski (CSP), being larger, deeper and located further from the main tourist trail, showed a slower but still substantial decline. In both lakes, large bodied cladocerans were rapidly eliminated, most likely as a result of intense fish predation (Gliwicz, 1985). In contrast, the largest and deepest lake, Wielki Staw Polski (WSP), has retained both high water transparency and large-bodied zooplankton species to the present day. This pattern suggests either lower fish densities or effective ecological buffering via the availability of offshore or deep-water refuge sites in WSP.

Comparable transformations have been documented in fish-stocked mountain lakes worldwide. In most cases, fish introductions have restructured plankton communities, with large-bodied crustaceans being replaced by smaller species and rotifers, accompanied by more or less pronounced changes in phytoplankton structure and biomass (Schindler *et al.*, 2001; Knapp *et al.*, 2005; Tiberti *et al.*, 2014). Shallow lakes tend to be more susceptible to fish-induced trophic shifts, whereas deeper systems may exhibit greater resistance—particularly when fish densities remain low or when compensatory mechanisms are in place (Tiberti *et al.*, 2014).

In some regions, active management has included the removal of introduced fish to restore native communities and reduce trophic status (Knapp *et al.*, 2001; Knapp and Sarnelle, 2008; Tiberti *et al.*, 2021). Most successful eradications of alien fish populations have been carried out in relatively shallow mountain lakes (Knapp *et al.*, 2005; Tiberti *et al.*, 2021). Despite growing awareness of these impacts, documented in a large number of studies reviewed in Ventura *et al.* (Ventura *et al.*, 2017), the cascading effects of fish introduction and removal on lake trophic states are still poorly understood and largely unpredictable. While some ecological shifts may be reversible over the short-term, others—particularly those involving the extinction of local populations of large-bodied zooplankton—may be more persistent (Sarnelle and Knapp, 2004; Knapp *et al.*, 2005). Understanding the trajectories and mechanisms of such recovery is crucial for evaluating the effectiveness of ecological restoration in these systems.

This study aims to assess long-term changes in the zooplankton community structure and trophic state in three relatively deep alpine-type lakes of the Five Lakes Valley—PSP, CSP and WSP—all of which were historically stocked with fish. We pay special attention to the ecological consequences of recent fish population reduction efforts in PSP, the lake most affected by tourism and eutrophication.

We hypothesized that fish removal or substantial reduction in the PSP would lead to:

- A recovery of fish-predation-sensitive, large-bodied cladocerans (*Daphnia pulicaria, Holopedium gibberum* and *Polyphemus pediculus*) that survived in neighboring WSP, where fish densities remained low, whereas these taxa were extirpated from CSP and PSP under high fish densities.
- A shift in the vertical distribution of large-bodied zooplankton toward shallower depths during the day due to reduced predation risk in PSP.
- A decrease in phytoplankton biomass in PSP resulting from enhanced zooplankton grazing pressure and reduced nutrient input by fish, with values moving away from those observed in CSP (high biomass under strong fish pressure) and approaching levels typical of WSP.
- Improved water transparency in PSP as a consequence of lower algal density, with clarity expected to increase toward the levels observed in WSP and distinctly exceed those in CSP.

To evaluate these expectations, we analyzed historical and recent data on water clarity, zooplankton composition and phytoplankton biomass across the three lakes.

## MATERIALS AND METHODS

### Study sites

We studied three neighboring lakes located in the Five Lakes Valley (Fig. 1). The valley is located within the granitic crystalline core of the High Tatras ridge, composed predominantly of granitoids, including pegmatite, aplite and mylonite. The valley floor is filled with Quaternary postglacial sediments (Michalik, 1951). All three lakes are situated above the timberline, and are of postglacial origin. Their morphometric and chemical characteristics are summarized in Table I. The smallest of the three—PSP—and the medium-sized CSP both drain into the largest lake, WSP, which subsequently outflows into the Roztoka River via a 65-m-high waterfall. This natural barrier has likely prevented post-glacial upstream fish colonization for millennia.

**Table I:** Morphometric characteristics and chemical parameters of the studied lakes based on published data.

	PSP	CSP	WSP
Latitude	49°12′45″N	49°12′17″N	49°12′33″N
Longitude	20° 20′80″E	20°01′36″E	20°02′27″E
Altitude (m a.s.l)^a^	1669	1722	1665
Area (ha)^a^	7.7	12.7	34.1
Catchment area (ha)	103	61	585
Max depth (m)^a^	34.6	50.4	79.3
Mean dept(m)^a^	14.6	22.2	37.7
Volume (10^6^ m^3^)^a^	1.13	2.826	12.967
pH^b^	7.23	6.73	7.03
TP (μmol/L)^b^	0.158	0.097	0.106
TON (μmol/L)^b^	10.8	8.8	8.1
Chlorophyll-*a* (μg/L)^b^	8.4	4	0.9
Fish introduction/reproduction since^c^	1960/1970	1960/1970	1980/?

According to:

^a^Jańczak (Jańczak, 1999),

^b^Kopáček *et al.* (Kopáček *et al.*, 2006),

^c^Gliwicz (Gliwicz, 1986).

Chemical data originate from single-point measurements.

There are two additional water bodies in the valley, but were not included in our study: Mały Staw Polski (MSP), a small and shallow pond (1.2 m deep) that has been stocked with fish; and Zadni Staw Polski (ZSP), the most remote and relatively deep lake in the valley (31.6 m deep), which remains fishless to this day.

### Fish populations and removal

All lakes in the valley remained fishless until the mid-20th century. Detailed information on the timing, extent and species composition of anthropogenic fish introductions is largely unavailable. Following the establishment of Tatra National Park in 1955, fish introduction became illegal, and any prior stocking activities—likely carried out by private individuals—were never formally documented. As a result, various sources propose differing timelines for the first appearance of fish in these lakes; in this study, we follow the chronology proposed by Gliwicz (Gliwicz, 1986) (Table I). At present, self-sustaining populations of brook trout (*Salvelinus fontinalis*) occur in PSP, WSP, CSP and MSP, while brown trout (*Salmo trutta*) is confined to PSP (authors’ unpublished data).

In accordance with the Tatra National Park’s decision to control and reduce fish populations in the PSP, we conducted targeted fish removal sessions during short field expeditions between 2021 and 2023. Fish control was carried out by gill netting and rod-angling in the littoral area, and by gillnetting alone in the pelagic zone. Nordic-type benthic gillnets used in the littoral (hereafter referred to as littoral gillnets) consisted of 12 contiguous panels with 12 mesh sizes arranged in a geometric series from 5 to 55 mm (CEN, 2015). Each mesh panel was 1.5 m high and 2.5 m long. The combined multi mesh net was 1.5 m high and 30 m long. Eleven littoral gillnets were randomly distributed throughout the entire littoral zone of the lake, set parallel to the shoreline at depths not exceeding 6 m. Three pelagic Nordic-type gillnets (each consisting of three 27.5 m long, 6 m high panels, with 11 mesh sizes ranging from 6.25 to 55 mm) were deployed stepwise from the surface down to a depth of 18 m in randomly selected areas of the pelagic zone. An additional gillnet with a mesh size of 24 mm (knot to knot), measuring 50 m in length and 6 m in height, was also deployed in the subsurface pelagic zone.

Fish sampling in PSP began several days after ice-off, typically in the second half of June, and was conducted over a period of 4–6 days (Supplementary Table S1). A second sampling season took place prior to ice formation, usually in mid-October. Depending on prevailing weather conditions, autumn sampling lasted between 3 and 6 days each year. The number of littoral and pelagic gillnets, as well as the duration of trout sampling, varied among seasons. Gillnetting effort—defined as the number of nets of a given type multiplied by the number of sampling nights—ranged from 24 to 108 for littoral gillnets and from 8 to 36 for pelagic gillnets across seasons. All gillnets were deployed in the evening and retrieved the following morning, with an approximate soak time of 12 h.

Captured fish were identified to species, counted and weighed to the nearest 1 g, separately for each gear type. Catch-per-unit-effort (CPUE) and weight-per-unit-effort were calculated for each gear category (littoral and pelagic), standardized to 100 m^2^ of net area deployed for 12 h.

Simultaneously, shoreline angling was conducted using fly and spinning techniques. All angling catches were similarly identified, counted and weighed. In addition, fish from one pelagic net and three littoral nets were individually measured for total length (to the nearest 0.5 cm) and weight (to the nearest 1 g).

The decline in CPUE between 2021 and 2023 in both the pelagic and littoral zones for both fish species, as well as the number of individuals removed during this period, was used to estimate fish abundance at the beginning of 2021 and the end of 2023 (Williams *et al.*, 2002). Accurate population size estimates from removal studies rely on minimally adequate capture probabilities and initial population size (Pine *et al.*, 2003).

We applied the following formula:


$$\mathbf{E}=\mathbf{CPU}{\mathbf{E}}_{\mathbf{2021}}/\mathbf{CPU}{\mathbf{E}}_{\mathbf{2023}}$$

$${\mathbf{N}}_{\mathbf{2021}}={\mathbf{N}}_{\mathbf{2023}}+\mathrm{Y},\mathrm{where}\;{\mathrm{N}}_{\mathbf{2023}}={\mathbf{N}}_{\mathbf{2021}}/\mathbf{E}$$

Where:

(i) E represents the change in fishing efficiency, assumed to result from a change in fish density.
(ii) Y is the number of fish removed between autumn 2021 and autumn 2023.
(iii) N_2021_ is the estimated number of fish present in the lake before harvesting in 2021.
(iv) N_2023_ is the estimated number of fish remaining in the lake at the end of 2023.

This approach assumes that CPUE is linearly related to fish density and that other factors affecting catch efficiency remained constant throughout the study period.

### Fish-mediated phosphorus retention

To estimate P, the seasonal phosphorus load attributable to fish activity in the ice-free period and normalized to lake surface area, we applied the following formula:


$$\mathrm{P}=\mathrm{Fd}\times{\mathrm{P}}_1,$$

where Fd represents fish density (fish/m^2^), calculated as estimated number of fish in the lake (N_2021_ or N_2023_) divided per lake area, and P_1_ is the estimated seasonal phosphorus input per fish (1100 mg P/fish/ice-free season), based on calculations by Dawidowicz (1985) for a neighboring Tatra lake, where airborne insects constitute the main summer food source for salmonid fish.

The estimated phosphorus input was compared to the maximum annual phosphorus load for oligotrophic lakes (125 mg/m^2^) as defined by Vollenweider (Vollenweider, 1976), applying a depth-based correction using the mean depth of PSP (14.6 m) to assess the potential influence of fish-derived nutrient enrichment.

### Physical parameters and plankton communities

To assess long-term changes associated with fish introductions and removal, we compared historical and recent data on water transparency and the taxonomic composition of pelagic mesozooplankton in the studied lakes over a time span of more than a century. Historical data (1881–1985) on Secchi disk depth and zooplankton composition were obtained from Gliwicz (Gliwicz, 1985) and Woźniczka (Woźniczka, 1964), who compiled and cited original records from earlier researchers alongside their own observations. Contemporary data (2012–2023) were collected as part of our field surveys. Although fieldwork occurred across different seasons during this period, for the purposes of temporal comparison, we used here only data collected during the summer stratification period (late July—early September).

Physical water parameters and zooplankton samples (2012–2023) were collected from an anchored dinghy positioned near the deepest point of each lake. These surveys included vertical profiling of temperature, dissolved oxygen and chlorophyll *a* (used as a proxy for phytoplankton biomass), measured using a submersible YSI EXO1 multiparameter probe (YSI Inc., USA). Water transparency was measured using a plain white, circular Secchi disk with a diameter of 25.5 cm.

Morphometric and chemical characteristics of the lakes were compiled from published sources (Table I).

Planktonic animals were collected by vertically towing a quantitative Apstein plankton net from the lake bottom to the surface using a graduated line. The net had a circular inlet with a diameter of 14 cm and a mesh size of 50 μm. Zooplankton samples were preserved in either 4% formalin (Chempur, Poland) or, after water removal, in 96% ethanol (Chempur, Poland), and subsequently identified and quantified using a Nikon SMZ1000 (Nikon, Japan) stereomicroscope. Crustaceans were analyzed quantitatively during 2012–2023, whereas rotifer communities were assessed qualitatively during 2021–2023. Rotifers were not included in the quantitative analyses because the 50 μm mesh size was not optimal for reliably estimating their abundance. The rotifer species are presented in Supplementary Table S7.

Vertical distribution of zooplankton in PSP was assessed on three occasions using a quantitative closing Apstein plankton net (150 μm mesh size, 28 cm diameter circular opening). Samples were collected from discrete 6-m water layers, with two replicate samples taken from each stratum. The total filtration rate of the *Daphnia* population in PSP was estimated using the relationship between individual filtering rate and ambient temperature reported by Mourelatos and Lacroix (Mourelatos and Lacroix, 1990), combined with our own data on *Daphnia* density and lake water temperature collected in 2023. This parameter was derived to assess whether changes in *Daphnia* population density following variation in fish density could account for observed changes in phytoplankton abundance during this period, given that daphnids are the primary phytoplanktivores in the lake.

## RESULTS

### Fish removal

Between 2021 and 2023, a total of 5453 fish (>50 mm) were removed from PSP, yielding a combined biomass of 655.7 kg—equivalent to a catch yield of 85.2 kg/ha. Two species were recorded in the catch. Brown trout (*S. trutta*) was the dominant species, accounting for 4762 individuals and 575.9 kg of biomass. Brook trout (*S. fontinalis*) was less abundant, with 691 individuals and a total biomass of 79.8 kg. The highest catch numbers were recorded during the first year of the removal effort, with 3053 fish caught and a total biomass of 383.3 kg. A detailed summary of catch data by gear type and year is presented in Supplementary Table S2.

Estimates of population abundance for both trout species were obtained using the decline in CPUE between 2021 and 2023, combined with data on the number of individuals removed by littoral and pelagic nets for both fish species. In the third year of the study, the effectiveness of gillnet sampling (CPUE) for brown trout decreased 3-fold by littoral net and 10-fold by pelagic net compared to the first year of removals, while for brook trout the CPUE declined 5-fold by littoral nets and 11-fold by the pelagic nets (Supplementary Table S3).

**Fig. 2:**
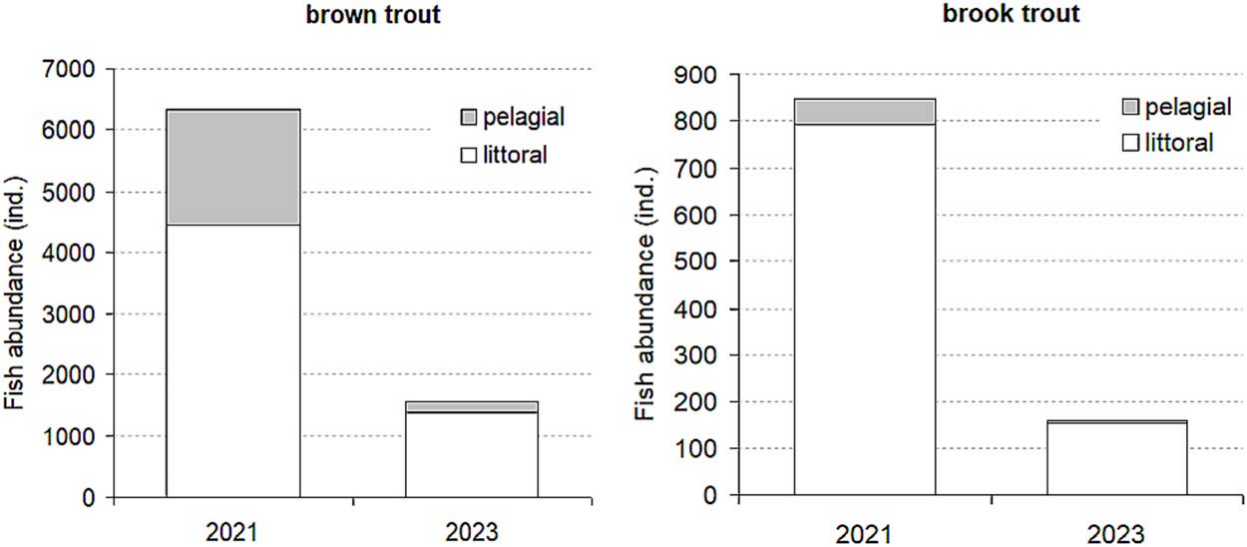
Estimated abundances of brown trout and brook trout in PSP Lake before and after fish removal, based on CPUE data and cumulative catch records.

Based on the observed decline in CPUE over time and cumulative fish removal data, the estimated number of brown trout in PSP at the beginning of the study in 2021 was 6330 individuals, and 848 for brook trout, yielding a total density of 932 fish (>50 mm) per hectare. By the end of 2023, following targeted removal efforts (excluding natural reproduction), the populations declined to 1568 brown trout and 158 brook trout, corresponding to a total density of 224 fish per hectare (Fig. 2, Supplementary Table S4).

### Fish size structure

The total length of captured fish ranged from 48 to 357 mm, and body mass ranged from 1.04 to 352 g. Among brown trout (*S. trutta*), individuals measuring 240–280 mm were most common. In the final year of the study, the proportion of smaller individuals (115–140 mm) increased notably (Supplementary Fig. S1, Fig. S2).

### Fish-mediated phosphorus retention

Given the estimated trout densities in PSP (0.093 ind/m^2^ in 2021 and 0.022 ind/m^2^ in 2023; see Supplementary Table S4), and assuming a per capita annual organic phosphorus input via consumption and mineralization of airborne insects of 1100 mg/m^2^/individual/season (after Dawidowicz, 1985), the total phosphorus input from this source was estimated at ~103 mg/m^2^/season in 2021 and 25 mg/m^2^/season in 2023.

### Water transparency

**Fig. 3:**
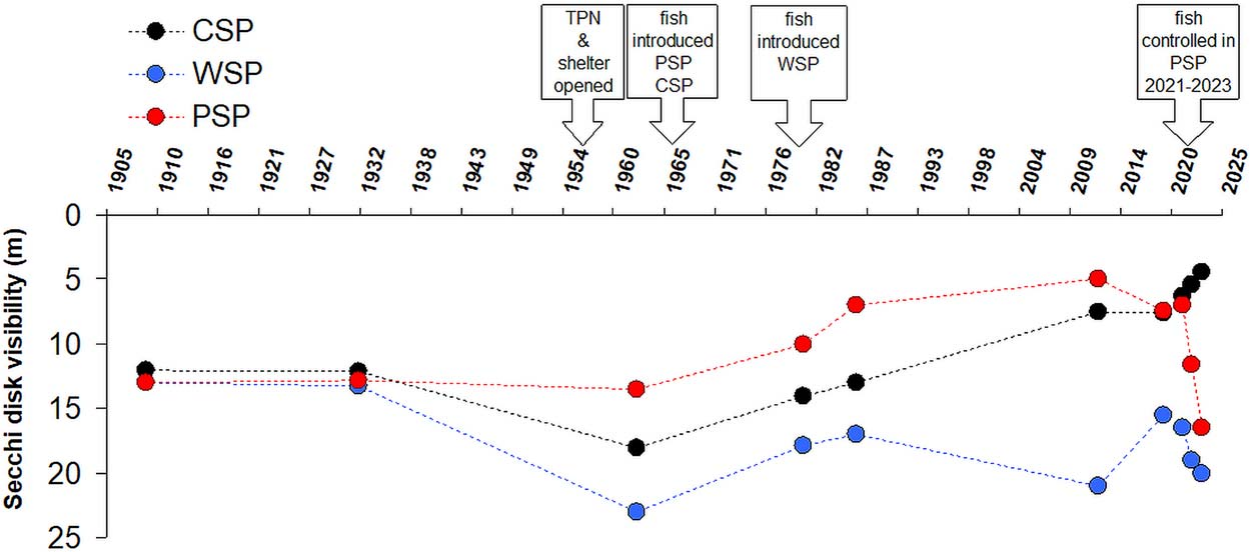
Secchi disk visibility over time in three lakes of the Five Lakes Valley: Przedni Staw Polski (PSP), Czarny Staw Polski (CSP) and Wielki Staw Polski (WSP).

The earliest records of water transparency (measured via Secchi disk visibility) in the studied lakes date back to the early 20th century, when all three lakes were fishless. At that time, summer transparency was similar across the lakes, averaging ~12–13 m (Fig. 3). In the early 1960s, following the establishment of a national park, water clarity improved in WSP to 17–23 m, and in CSP to 15–18 m, while remaining relatively stable in PSP at 13–13.5 m (Fig. 3).

**Fig. 4:**
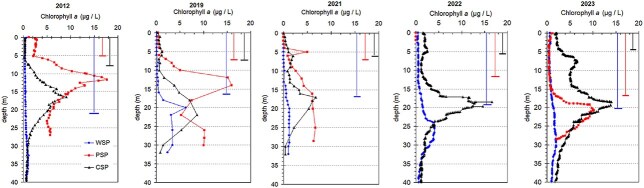
Vertical distribution of chlorophyll *a* and Secchi disk visibility in the studied lakes—PSP, CSP and WSP—during selected years from the 2012–2023 period. Note: chlorophyll *a* data for PSP are partly unavailable for 2022.

Soon after, fish were introduced into PSP and CSP in the mid-1960s, with established populations evident by 1970 (Gliwicz, 1986). Following these introductions, water transparency gradually declined in both lakes, reaching recent levels of 5–7 m. In contrast, WSP—where fish introduction began in the 1980s but did not result in a dense or self-sustaining population—has maintained high water clarity, fluctuating between 17 and 23 m.

Between 2021 and 2023, fish removal efforts in PSP were associated with a rapid and substantial increase in water transparency, from 7 m in 2021 to 16.5 m in 2023—a value not previously recorded. Conversely, in CSP, where no fish removal took place and fish density remained high, transparency declined over the same period from 6.3 to 4.4 m. In WSP, where fish density has remained low and unchanged, transparency increased modestly from 16.5 m in 2021 to 20 m in 2023.

### Phytoplankton biomass patterns and depth distribution (2012–2023)

Among the three surveyed lakes, the highest total phytoplankton biomass—estimated using its proxy, the area under the chlorophyll *a* vertical distribution curve—was recorded in PSP prior to fish removal (Fig. 4, Supplementary Table S5). Following the reduction in fish abundance, PSP exhibited a gradual decline in phytoplankton biomass, and by 2023, its biomass levels had fallen below those observed in CSP. Throughout the study period, WSP consistently exhibited the lowest proxy values of phytoplankton biomass among the three lakes (Supplementary Table S5).

In addition to changes in total biomass, PSP showed a marked shift in the vertical distribution of phytoplankton. By 2023, the depth of peak biomass in PSP had moved from 12–14 m to ~20 m—suggesting a substantial deepening of the phytoplankton maximum. In contrast, no such pronounced changes in vertical distribution were observed in CSP or WSP during the same period (Fig. 4).

### Zooplankton community structure and density

The introduction of fish into the lakes of the Five Lakes Valley caused major shifts in zooplankton community structure. In PSP, large-bodied cladocerans—including representatives of the *Daphnia pulex* group (*Daphnia tenebrosa* or *Daphnia pulicaria*), *H. gibberum* and *P. pediculus*—disappeared shortly after fish establishment. These taxa were replaced by members of the *Daphnia longispina* group, most closely resembling *Daphnia galeata* in morphology (Table II). The copepod *Cyclops abyssorum tatricus* became the dominant mesozooplankton species.

**Table II:** Composition of the pelagic mesozooplankton community in the three studied lakes of the Five Lakes Valley during the last century, under conditions of fish absence and presence at low or high densities

	PSP					CSP					WSP				
Fish density	Absent^b^	Low^b^	High^b^	High^d^	Low^d^	Absent^b^	Low^b^	High^b^	High^d^	High^d^	Absent^b^	Absent^b^	Low^b^	Low^d^	Low^d^
	1881–1909^a^	1962–1963^a^	1973–1983^a^	2012–2019^d^	2021––2023^d^	1881–1909^c^	1962–1963^c^	1973–1983^d^	2012–2019^d^	2021–2023^d^	1881–1909^a^	1962–1963^a^	1973–1983^a^	2012–2019^d^	2021–2023^d^
*Holopedium gibberum*	+	−	+	−	−	+	+	−	−	−	+	+	+	+	+
*Daphnia pulex* group	+	+	−	−	−	+	+	−	−	−	+	+	+	+	+
*Daphnia longispina* group	−	+	−	+	+	−	+	−	−	+	−	+	−	−	−
*Polyphemus pediculus*	+	−	−	−	−	+	−	−	−	−	+	+	−	+	+
*Diaptomus graciloides*	−	−	−	−	−	−	−	−	−	−	+	−	−	−	−
*Cyclops abyssorum tatricus*	+	+	+	+	+	+	+	+	+	+	+	+	+	+	+

According to:

^a^Gliwicz (Gliwicz, 1985),

^b^Gliwicz (Gliwicz, 1986),

^c^Woźniczka (Woźniczka, 1964),

^d^Present study.

**Fig. 5:**
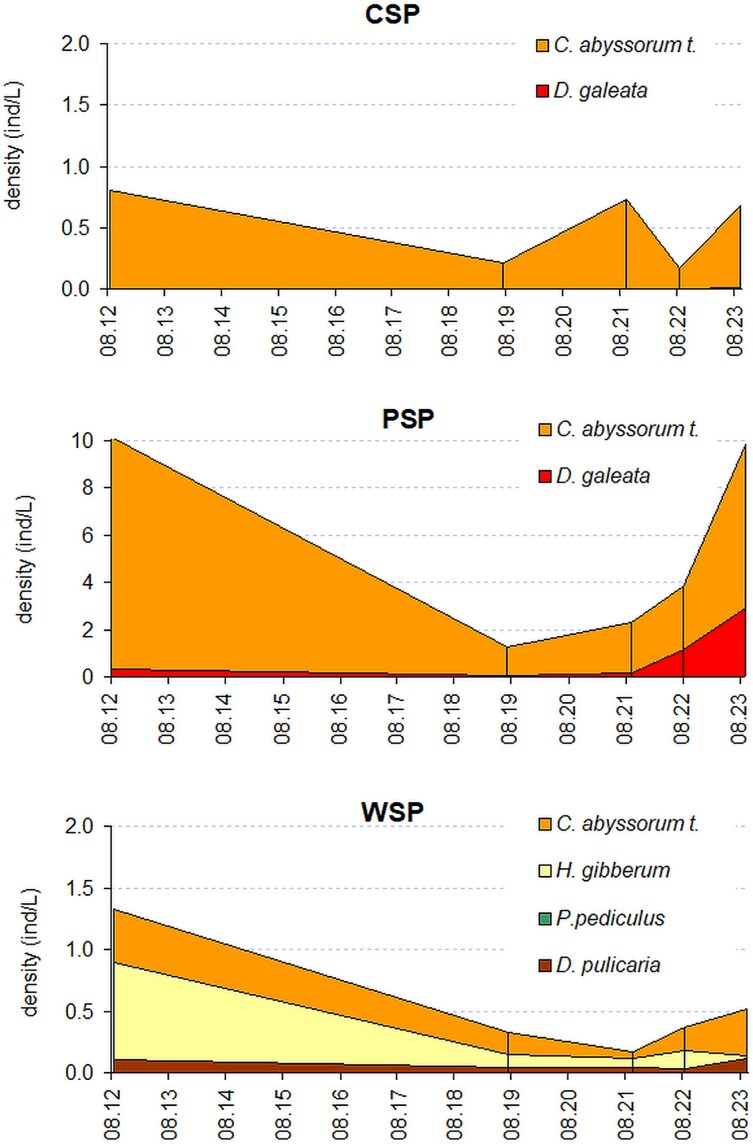
Composition of pelagic mesozooplankton in the studied lakes from 2012 to 2023: PSP, CSP and WSP. Note the different scales on the Y-axes.

Since the onset of fish reduction efforts in PSP in 2021, an increase in both the abundance and proportional dominance of *D. galeata* has been observed (Fig. 5, Supplementary Table S6). However, by 2023, no signs of recolonization by large-bodied cladocerans from the *Daphnia pulex* group or by *H. gibberum*—both of which persist in the neighboring, fish-poor WSP located only 100 m away—had been detected in PSP (Fig. 5, Supplementary Table S6).

In CSP, the mesozooplankton community became severely impoverished following fish introduction. Large cladocerans—including *Daphnia* from the *D. pulex* group, *H. gibberum* and *P. pediculus*—disappeared and have not been recorded for several decades (Table II). The current assemblage is numerically dominated by the copepod *C. abyssorum tatricus*, while *D. galeata* is detected only sporadically and in low densities (Fig. 5, Supplementary Table S6).

In contrast to the other two lakes, fish introduction in WSP did not cause substantial changes in zooplankton community composition (Table II). The pelagic assemblage continues to be dominated by large-bodied cladocerans, including *H. gibberum* and members of the *D. pulex* group, alongside *C. abyssorum tatricus. Polyphemus pediculus* was observed only occasionally and at low densities (Fig. 5, Supplementary Table S6).

### Vertical distribution of mesozooplankton in PSP (2021–2023)

**Fig. 6:**
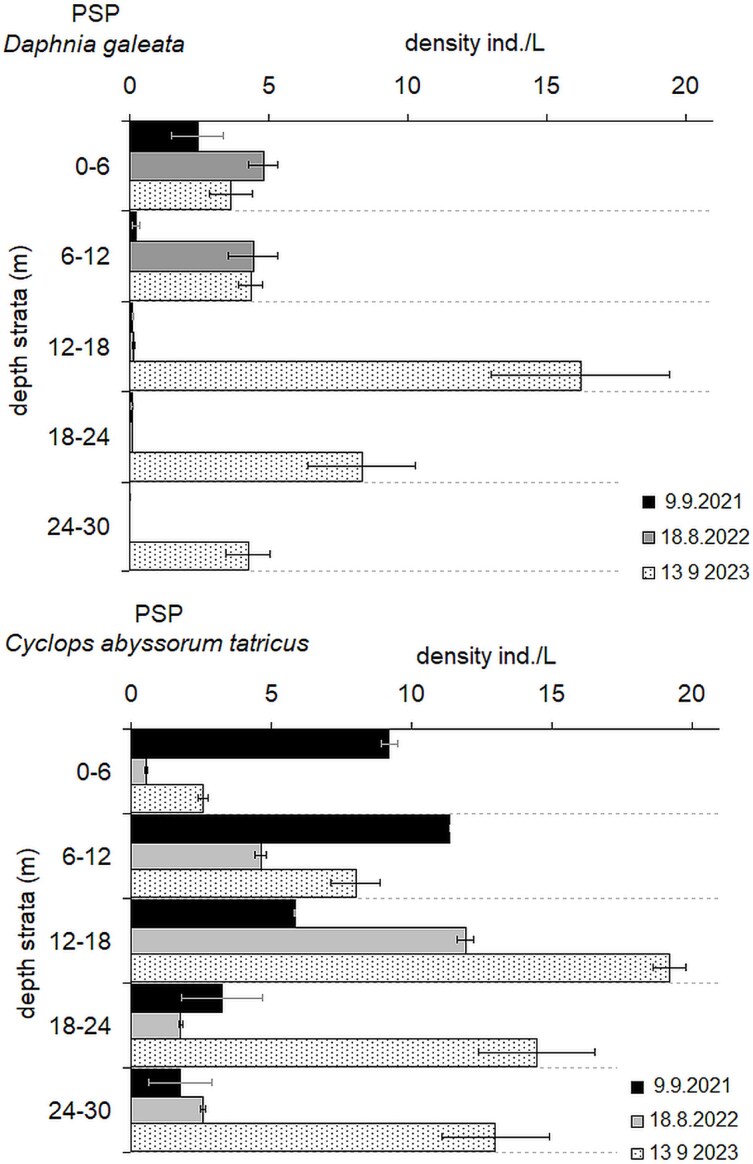
Changes in the vertical distribution of *Daphnia galeata* and *Cyclops abyssorum tatricus* in PSP over time (mean ± SE).

During daytime hours, *D. galeata* occupied the upper water layers of PSP in 2021 and 2022, when fish densities were high or moderate (Fig. 6). The mean depth of the *D. galeata* population was 4.6 m in 2021 and 6.2 m in 2022. By 2023, following a substantial reduction in fish abundance, *Daphnia* shifted to deeper waters, reaching a mean depth of 15.9 m.

Meanwhile, *C. abyssorum* exhibited a progressively deeper and broader vertical distribution. Its mean depth increased from 10.6 m in 2021 to 15.3 m in 2022, and further to 17.9 m in 2023.

## DISCUSSION

### Long-term effects of fish introduction on zooplankton and lake trophic status

Gliwicz’s early hypothesis (Gliwicz, 1963) that fish introductions would reshape zooplankton communities and promote eutrophication in Tatra mountain lakes has since been corroborated by long-term changes observed in the Five Lakes Valley (Gliwicz, 1985, present study). Within a few years of trout stocking, large-bodied cladocerans were no longer detected in two of the three lakes (PSP and CSP), i.e. those with higher fish densities. In the absence of large and efficient grazers, smaller cladocerans increased in relative abundance (Table II). *Cyclops abyssorum tatricus*, a copepod capable of passing eggs unharmed through fish guts (Gliwicz and Rowan, 1984), also persisted despite fish predation.

A pronounced decline in water transparency followed fish introduction—first in PSP, a smaller lake strongly affected by tourism activity, and later in the larger, more remote CSP (Fig. 3). The mechanisms underlying this decline remain unclear. It is uncertain whether the disappearance of large grazers directly promoted phytoplankton growth, or whether trout enhanced nutrient cycling, thereby stimulating primary production. Both mechanisms may have contributed to the observed trophic shifts.

### Mechanisms driving eutrophication after fish introduction

Fish are known to act as biological traps for organic matter and efficient mineralizers, releasing nutrients into the water via fecal excretion and across body surfaces (Sereda *et al.*, 2008). During the ice-free period, trout feed extensively in epilimnion on airborne terrestrial invertebrates, primarily insects, that fall onto the lake surface; in autumn and winter, their diet shifts toward benthic chironomids and zooplankton. These feeding patterns have been documented in a neighboring Tatra lake (Dawidowicz and Gliwicz, 1983) and in the present study in PSP (Supplementary Fig. S3).

In contrast, fishless lakes receive fewer airborne invertebrates (Rolla *et al.*, 2018) and export more nutrients through the emergence of adult aquatic insects (Pope *et al.*, 2009; Piovia-Scott *et al.*, 2016). These differences likely constrain phytoplankton productivity and help maintain low trophic status in naturally fishless systems.

Paleolimnological analysis of sediment cores from PSP and WSP (Sienkiewicz and Gąsiorowski, 2016) estimated a 2-fold increase in total phosphorus concentration by 2010. No analogous data are available for CSP according to our knowledge.

### Fish removal in PSP: effectiveness and limnological response

PSP showed the fastest decline in water transparency among the three lakes, dropping from ~ 15 m to 5–7 m within a few years of fish introduction (Fig. 3). By 2021, trout densities reached 932 fish/ha—relatively high, although not extreme by alpine lake standards (cf. Tiberti *et al.*, 2021). PSP also faces practical concerns, as it supplies drinking and sanitary water to a popular mountain shelter. Elevated turbidity linked to fish presence increases water treatment needs. Despite the presence of a wastewater facility, high visitor numbers and human activity likely increase eutrophication pressure, reinforcing the need for ecological intervention.

Our fish removal efforts, carried out over three consecutive summer seasons, resulted in a 4-fold reduction in fish abundance. Based on the number of fish removed during the study period, we estimate that by the end of 2023, ~1569 brown trout (>50 mm) and 157 brook trout (>50 mm) remained in the lake, corresponding to a total density of 224 fish per hectare (Fig. 2).

Our effort proved insufficient to achieve complete fish removal; nevertheless, it demonstrated that gillnetting can rapidly reduce fish populations and, if implemented at higher intensity, may have the potential to eradicate fish entirely. Gillnetting-based removal has proven successful in several previously published cases; however, these efforts were typically more intensive and conducted in lakes shallower than PSP (Knapp *et al.*, 2001; Knapp and Sarnelle, 2008; Tiberti *et al.*, 2021). Complete eradication becomes increasingly challenging in deeper and larger systems (Knapp and Matthews, 1998; Pacas and Taylor, 2015). In such cases, chemical methods—such as rotenone application—have been successfully used to eradicate fish populations (McClay, 2005). However, because PSP serves as a source of drinking water for the nearby mountain shelter, the use of chemical eradication techniques was deemed unacceptable.

### Mechanisms of water transparency recovery

Despite incomplete fish eradication, water transparency improved remarkably in PSP. Secchi disk visibility increased from 7 to 16.5 m between 2021 and 2023 (Fig. 3), representing a greater gain than reported in most lake restoration studies to date. In most oligotrophic mountain lakes shallower than PSP, the effects of fish eradication on water transparency and phytoplankton abundance have been negligible or weak (Parker *et al.*, 2001; Tiberti *et al.*, 2019), although some notable exceptions have been reported (Parker and Schindler, 2006). In that study, an increase in water transparency from 3 to 9 m following fish removal was attributed to enhanced filtration of mineral seston by large-bodied *Daphnia*, which reappeared and increased in abundance following fish removal. This raises the question of which mechanisms could explain the pronounced increase in water transparency observed in PSP between 2021 and 2023. Notably, no major shifts in zooplankton community composition were detected during this period in PSP (Fig. 5). Large-bodied cladocerans did not recolonize the lake, and although the density of the smaller *D. galeata* increased several-fold, its abundance seems insufficient to exert the level of grazing pressure necessary to substantial “top-down” reduction of phytoplankton biomass. An estimate of the total *Daphnia* filtering rate (calculated according to Mourelatos and Lacroix, 1990) at the population densities achieved in PSP after fish removal in 2023 was only about 4 mL/L/day—which seems far too low to cause the observed increase in water transparency.

An alternative explanation for the observed increase in water transparency during the fish control period is a bottom-up effect on phytoplankton abundance driven by reduced nutrient input following fish removal. Although we did not measure phosphorus concentrations in the water before and after the intervention, our rough estimates indicate that, prior to 2021, phosphorus loading in PSP due to fish excretion alone amounted to ~103 mg/m^2^/ice-free season. This value approaches the upper limit for oligotrophic lakes as defined by Vollenweider (Vollenweider, 1976)—125 mg/m^2^/year. Such elevated nutrient input likely contributed to historically low water transparency and elevated phytoplankton biomass. Following the decline in fish populations, phosphorus input dropped ~4-fold—falling well below the Vollenweider threshold—and coincided with a substantial recovery of water clarity in PSP.

While fish reduction limited nutrient inputs to surface waters, summer thermal stratification in this relatively deep mountain lake may have restricted the upward flux of phosphorus accumulated in the hypolimnion during decades of fish-mediated enrichment. Such stratification-driven isolation of hypolimnetic phosphorus may not occur in shallow, polymictic lakes after fish removal, where accumulated nutrients are more readily returned to surface waters through vertical mixing. This mechanism may explain the observed shift toward clearer surface waters, accompanied by a redistribution of phytoplankton biomass to deeper strata in PSP—a pattern not reported in previous studies of other lakes. Over a prolonged period of reduced fish density or complete fish eradication, phytoplankton biomass throughout the water column in PSP may ultimately decline, as natural nutrient export would exceed diminished or absent fish-mediated nutrient inputs.

In contrast, in CSP, where fish densities remained high throughout our study, no improvement in water transparency was observed; on the contrary, transparency declined even further—from 7.3 m in 2021 to 4.4 m in 2023 (Fig. 3).

### Vertical distribution of zooplankton in relation to water transparency

In PSP, reduced fish predation ultimately altered the vertical distribution of zooplankton. Following the decline in fish densities and concomitant increases in water transparency, *Daphnia* began occupying deeper strata (Fig. 6). While one might expect upward shifts in response to reduced predation risk, the opposite pattern suggests alternative mechanisms: individuals may have responded either to increased algal food availability at depth or to elevated ultraviolet (UV) radiation near the surface resulting from increased water transparency (Fig. 4), according to the mechanism proposed by Fischer *et al.* (Fischer *et al.*, 2015). The latter factor may also explain the concurrent downward shift of *C. abyssorum*, a species not directly dependent on phytoplankton. Throughout this period, oxygen concentrations remained high across the entire water column (Supplementary Fig. S4), ruling out oxygen limitation as a constraint on vertical distribution.

These findings support the transparency-regulator hypothesis (Williamson *et al.*, 2011), which posits that in subalpine lakes, the relative importance of UV avoidance increases with water transparency and can override antipredator responses. In this context, UV exposure may have become an increasingly important determinant of mesozooplankton vertical distribution in PSP.

### Comparison among lakes and management implications

In contrast to the two other lakes, WSP—the largest and deepest lake—continues to support large-bodied zooplankton and high water transparency despite the presence of trout. Although fish densities were not measured using CPUE, anectodal data, together with opportunistic observations from the shorelines, indicated far fewer fish than in PSP and CSP, a situation suggesting lower fish densities (Tiberti *et al.*, 2022).

The continued presence of large-bodied cladocerans in both WSP and the uppermost lake in the cascade—ZSP (author’s unpublished data), which remains fishless—represents a valuable source for the potential recolonization of these taxa in previously stocked lakes in the valley, provided that fish eradication efforts are ultimately successful.

However, as complete removal has not been achieved in PSP, the long-term ecological benefits of the intervention remain questionable. The remaining fish, benefiting from reduced competition and abundant food resources, may persist and reproduce, potentially reversing the progress made to date. Given the high fecundity of salmonids, such a rebound could quickly lead to rising fish densities and a renewed risk of eutrophication. We therefore strongly recommend that the Tatra National Park authorities continue—and ideally intensify—the fish removal program in PSP replicating successful eradication actions completed in other mountain ranges (Ventura *et al.*, 2017). To date, due to resource constraints, gillnets have been deployed only for a few days annually (Supplementary Table S1) whereas eradication efforts require much greater investment, involving continuous, year-round intensive gillnetting over several years (Knapp and Matthews, 1998; Pacas and Taylor, 2015). Furthermore, incorporating complementary techniques such as electrofishing may enhance overall efficiency by targeting smaller individuals that inhabit the littoral zone and are often not captured by gillnets alone (Tiberti *et al.*, 2021).

## CONCLUSIONS

High-mountain fishless lakes represent some of the last remaining pristine oligotrophic freshwater ecosystems, characterized by exceptionally low nutrient availability, unique biotic communities and high water transparency. Over the past decades, many such systems have experienced profound ecological alterations, sometimes including eutrophication, following fish introductions driven by short-sighted human decisions. Our study demonstrates that these introductions can profoundly alter zooplankton community composition and trophic dynamics, leading to long-term ecosystem degradation, as exemplified by the lakes in the Five Lakes Valley.

In PSP, partial fish removal led to a marked improvement in water transparency—an effect that likely reflects reduced nutrient input rather than enhanced zooplankton grazing. This suggests a potentially dominant role of bottom-up processes in regulating productivity in these ecosystems. However, the persistence of residual fish populations limits the long term sustainability of the achieved ecological benefits and hinders the establishment of long-term recovery dynamics that could allow the return of large-bodied zooplankton species extirpated by fish decades ago.

Achieving complete restoration of such altered systems will require more intensive and spatially comprehensive fish removal strategies.

## Supplementary Material

Supplementary_data_FLV_fbag016

## Data Availability

Data supporting the findings of this study are available from the corresponding author upon request.
