# Supplementary material for: Effects of fish stocking and removal on plankton communities and trophic status in deep alpine-type lakes of the Five Lakes Valley (Tatra Mountains): Bottom-up and top-down regulation in the ecology of offshore animals. The lifetime passion of Z.Maciej Gliwicz
Source: J Plankton Res. 2026 Mar 26;48(2):fbag016. doi: 10.1093/plankt/fbag016 (PMC13019441; doi:10.1093/plankt/fbag016)
Supplement: Supplementary_data_FLV_fbag016 [file supplementary_data_flv_fbag016.doc]

**Supplementary data.**

**Effects of Fish Stocking and Removal on Plankton Communities and Trophic Status in Deep Alpine-Type Lakes of the Five Lakes Valley (Tatra Mountains)**

M. Ślusarczyk, T. Brzeziński, P. Dawidowicz, A. Kapusta, T. Zwijacz-Kozica, T. Kuczyński, A. Ochocka, K. Kozłowski

Three studied lakes of the Five Lakes Valley:

Przedni Staw Polski (PSP)

Czarny Staw Polski (CSP)

Wielki Staw Polski (WSP)

Table S1. Timing of fish removal activities at PSP

| Year | Spring | Autumn |
| --- | --- | --- |
| 2021 | 15-18.06 | 9-14.10 |
| 2022 | 28.06-03.07 | 18-20.10 |
| 2023 | 27.06-02.07 | 17-21.10 |

Table S2. Fish removal results from PSP

| **Year** | **Method** | **Brown Trout (Number [pcs])** | **Brown Trout (Mass [g])** | **Brook Trout (Number [pcs])** | **Brook Trout (Mass [g])** |
| --- | --- | --- | --- | --- | --- |
| 2021 | Littoral net | 1390 | 174,649 | 281 | 31,928 |
|  | Pelagic net | 1339 | 170,706 | 43 | 6,011 |
|  | Angling | 156 | 25,142 | 79 | 10,759 |
| 2022 | Littoral net | 640 | 71,990 | 168 | 16,825 |
|  | Pelagic net | 272 | 38,245 | 5 | 730 |
|  | Angling | 96 | 14,797 | 28 | 3,960 |
| 2023 | Littoral net | 791 | 69,734 | 84 | 8,990 |
|  | Pelagic net | 76 | 10,257 | 2 | 440 |
|  | Angling | 2 | 338 | 1 | 175 |

Table S3. Net fishing efficiency expressed as catch-per-unit-effort (CPUE) and weight-per-unit-effort (WPUE) index values in PSP.

| Year | Method | Brown Trout | | Brook Trout | |
| --- | --- | --- | --- | --- | --- |
|  |  | CPUE  [mean ± SD] | WPUE  [mean ± SD] | CPUE  [mean ± SD] | WPUE  [mean ± SD] |
|  |  |  |  |  |  |
| 2021 | Littoral net | 29.4 ± 24.9 | 3668.9 ± 3578.7 | 5.2 ± 6.7 | 592.1 ± 746.7 |
|  | Pelagic net | 14.5 ± 12.3 | 1819.7 ± 1662.9 | 0.8 ± 1.4 | 95.9 ± 205.2 |
| 2022 | Littoral net | 17.3 ± 10.7 | 1950.9 ± 1546.7 | 4.7 ± 4.2 | 467.4 ± 442.1 |
|  | Pelagic net | 5.9 ± 5.1 | 804.5 ± 713.4 | 0.1 ± 0.2 | 18.9 ± 36.3 |
| 2023 | Littoral net | 9.1 ± 7.9 | 782.2 ± 925.5 | 1.0 ± 2.6 | 104.8 ± 315.1 |
|  | Pelagic net | 1.4 ± 2.3 | 188.9 ± 379.2 | 0.07 ± 0.2 | 12.8 ± 33.2 |

**Notes**:

- **CPUE** (individuals/100m^2^/12h)
- **WPUE** (g/100m^2^/12h)

Table S4. Estimated number of fish in PSP based on CPUE and fish removal data

|  | ***Salmo trutta m. fario*** | ***Salvelinus fontinalis*** |
| --- | --- | --- |
| **Initial number of fish 2021** |  |  |
| Litoral | 4,457 | 794 |
| Pelagial | 1,867 | 55 |
| **Fish removed 2021–2023** |  |  |
| Litoral | 3,075 | 641 |
| Pelagial | 1,687 | 50 |
| **Final number of fish 2023** |  |  |
| Litoral | 1,382 | 153 |
| Pelagial | 180 | 5 |

Table S5. Chlorophyll *a* concentration (µg/dm²) in the 0–30 m water column (area under the curve of chlorophyll volume concentration versus depth in Fig 4.)

|  | | | | | |
| --- | --- | --- | --- | --- | --- |
|  | **2012** | **2019** | **2021** | **2022** | **2023** |
| PSP | 96.2 | 211.1 | 115.9 | missing | 87.4 |
| CSP | 45.2 | 110.3 | 69.7 | 119.7 | 181.9 |
| WSP | 8.4 | 55.0 | 19.3 | 29.6 | 9.3 |

Table S6. Density of mesoplankton in the pelagic zone of the three studied lakes 2012-2023 of the Five Lakes Valley (Tatra Mountains): Przedni Staw Polski (PSP), Czarny Staw Polski (CSP), Wielki Staw Polski (WSP)

| lake | date | *Daphnia galeata* | *Daphnia pulicaria* | *Holopedium gibberum* | *Polyphemus pediculus* | *Cyclops abyssorum* |
| --- | --- | --- | --- | --- | --- | --- |
| PSP | 17.08.2012 | 0.31 | 0 | 0 | 0 | 9.83 |
| PSP | 25.07.2019 | 0.07 | 0 | 0 | 0 | 1.21 |
| PSP | 09.09.2021 | 0.16 | 0 | 0 | 0 | 2.38 |
| PSP | 18.08.2022 | 1.21 | 0 | 0 | 0 | 3.22 |
| PSP | 13.09.2023 | 2.84 | 0 | 0 | 0 | 6.63 |
|  |  |  |  |  |  |  |
| CSP | 18.08.2012 | 0 | 0 | 0 | 0 | 0.80 |
| CSP | 25.07.2019 | 0 | 0 | 0 | 0 | 0.22 |
| CSP | 09.09.2021 | 0 | 0 | 0 | 0 | 0.62 |
| CSP | 18.08.2022 | 0 | 0 | 0 | 0 | 0.17 |
| CSP | 13.09.2023 | 0.01 | 0 | 0 | 0 | 0.60 |
|  |  |  |  |  |  |  |
| WSP | 17.08.2012 | 0 | 0.11 | 0.79 | 0 | 0.43 |
| WSP | 24.07.2019 | 0 | 0.03 | 0.08 | 0.002 | 0.18 |
| WSP | 08.09.2021 | 0 | 0.04 | 0.08 | 0 | 0.06 |
| WSP | 17.08.2022 | 0 | 0.04 | 0.11 | 0.001 | 0.19 |
| WSP | 12.09.2023 | 0 | 0.13 | 0.04 | 0 | 0.38 |

Table S7. Rotifer species recorded in the pelagic zone of the studied lakes during 2021–2023.

PSP – Przedni Staw Polski. CSP – Czarny Staw Polski. WSP – Wielki Staw Polski.

Symbols: +++ dominant species. ++ subdominant species. + present. – absent.

|  | PSP | CSP | WSP |
| --- | --- | --- | --- |
| *Ascomorpha ecaudis* | - | - | + |
| *Asplanchna priodonta* | + | + | + |
| *Collotheca mutabilis* | + | - | - |
| *Collotheca pelagica* | - | - | + |
| *Colurella obtusa* | - | - | + |
| *Conochiloides dossuarius* | +++ | + | + |
| *Keratella hiemalis* | + | - | - |
| *Keratella quadrata* | ++ | + | ++ |
| *Keratella tecta* | - | - | + |
| *Keratella valga* | - | + | - |
| *Lecane flexilis* | + | - | - |
| *Polyarthra dolichoptera* | + | + | +++ |
| *Polyarthra longiremis* | - | + | - |
| *Polyarthra remata* | + | - | + |
| *Sinantherina semibullata* | - | ++ | - |
| *Synchaeta lakowitizana* | - | +++ | + |
| *Synchaeta sp.* | - | +++ | - |
| *other rotifers* | - | - | + |

Figure S1. Frequency distribution of total length (mm) for brown trout captured in PSP


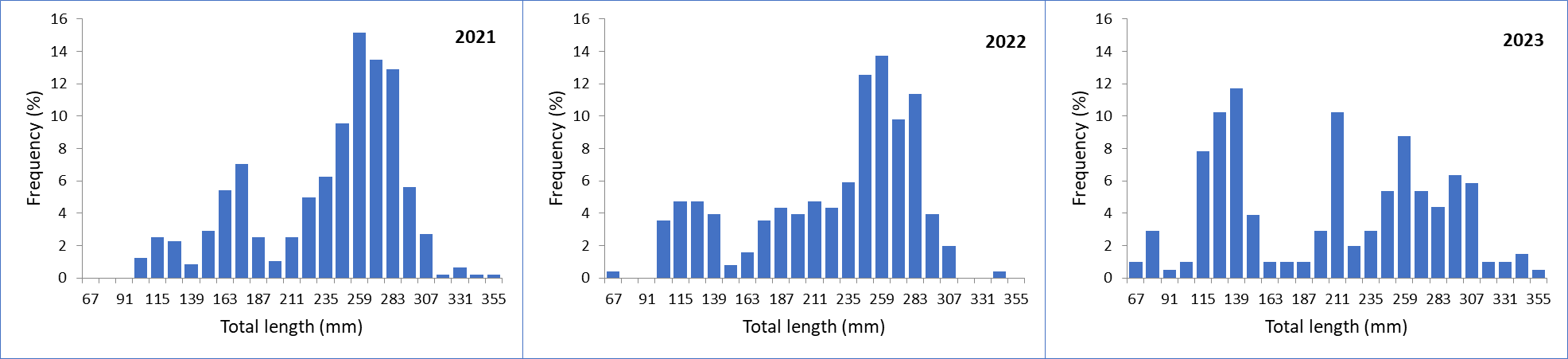


Figure S2. Frequency distribution of total length (mm) for brook trout captured in PSP


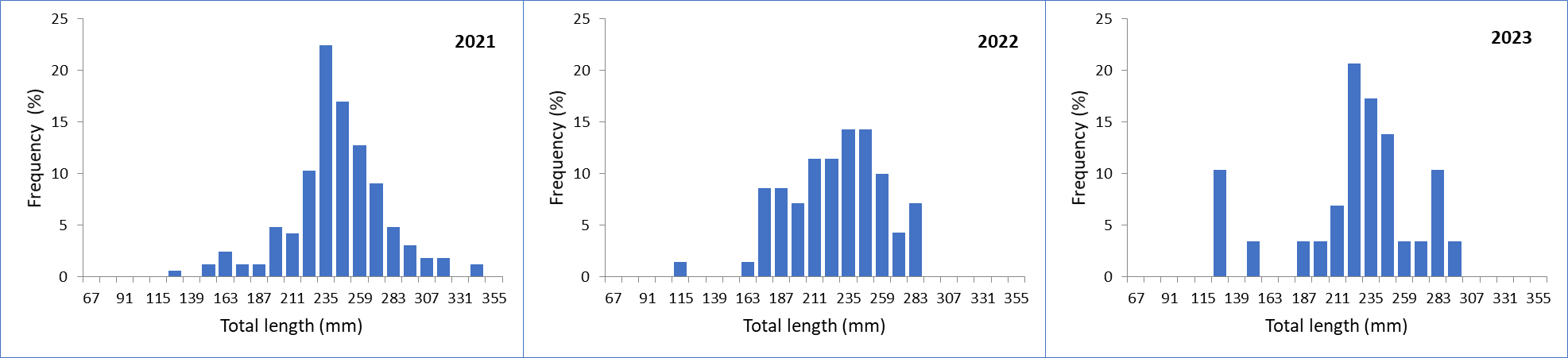


Figure S3. Seasonal variation in the average food composition of trouts collected in PSP. N denotes the number of individuals analyzed. Proportions are based on the numerical abundance of prey items. Green segments indicate terrestrial food sources.

June 2022

Octobre 2022

April 2023

N=84

N=130

N=24

Figure S4. Vertical profile of temperature and oxygen concentration in PSP in 2021 and 2023. Data for 2022 are not available.
